# Phosphorylated α-synuclein aggregated in Schwann cells exacerbates peripheral neuroinflammation and nerve dysfunction in Parkinson’s disease through TLR2/NF-κB pathway

**DOI:** 10.1038/s41420-021-00676-w

**Published:** 2021-10-12

**Authors:** Li Sun, Wen-Wen Jiang, Ye Wang, Yong-Sheng Yuan, Zhe Rong, Jing Wu, Yi Fan, Ming Lu, Ke-Zhong Zhang

**Affiliations:** 1 Department of Neurology, The First Affiliated Hospital of Nanjing Medical University, Nanjing, China; 2 Jiangsu Key Laboratory of Neurodegeneration, Department of Pharmacology, Nanjing Medical University, Nanjing, China

**Keywords:** Somatic system, Parkinson's disease

## Abstract

To investigate the mechanism of peripheral neuropathy in Parkinson’s disease (PD), we prepared a PD mice model by long-term exposure of 1-methyl-4-phenyl-1,2,3,6-tetrahydropyridine (MPTP) to mimic PD pathology in humans and the sciatic nerves were taken for further research. It turned out that phosphorylated α-synuclein (p-α-syn) was significantly deposited in Schwann cells (SCs) of sciatic nerves possibly contributing to degenerated myelin SCs and atrophied axons in MPTP group. Further analysis confirmed that toll-like receptors (TLRs) were implicated with PD peripheral neuropathy, in which TLR2 exhibits the predominant expression. Increased expression of inflammatory factors about TLR2/nuclear factor kappa-B (NF-κB) pathway was noted in MPTP group compared to saline group, with proteins on other pathways showing no changes. Moreover, MPTP-challenged mice exhibited worse motor ability and damaged nerve conduction, implicating that p-α-syn neurotoxicity might be relevant to impairments of motor and sensory nerves. After the treatment of CU-CPT22, a TLR2 antagonist, p-α-syn accumulation, motor and sensory function were ameliorated in CU-CPT22 combined with MPTP group. Thus, we demonstrated that pathological p-α-syn might combine TLR2 to affect SCs activation, inflammatory response as well as motor and sensory function through TLR2/nuclear factor kappa-B (NF-κB) signaling pathway. This study firstly demonstrates a novel mechanism of p-α-syn accumulated in SCs of peripheral nerves, which extends our understanding on SCs-mediated peripheral neuroinflammation related to TLR2/NF-κB signaling pathway and sheds light on potential new therapeutic avenues for PD.

## Introduction

Parkinson’s disease (PD) is a complicated progressive neurodegenerative disease characterized by loss of dopaminergic (DA) neurons in the substantia nigra pars compacta (SNpc) and over-activated glial cells, as well as the accumulation of α-synuclein (α-syn) [1, 2]. Phosphorylated α-syn (p-α-syn) has been reported to be prominent in PD, which enhances the toxicity of α-syn both in vivo and in vitro [1, 3, 4]. Recent researches revealed that except the central nervous system (CNS), p-α-syn exerts critical influence on the peripheral nervous system (PNS) during PD of early stage. It has been known that p-α-syn can be detected in dermal nerves, enteric nerves, autonomic nerves, mesenteric sympathetic ganglia, stellate ganglion, paravertebral sympathetic ganglia and epicardial plexus in PD onset [5–9].

The abnormal p-α-syn deposition evokes systemic inflammatory response, which is a trigger for PD and exacerbates subsequent DA degeneration [10]. Lipopolysaccharide of low doses in mice induced the pro-inflammatory profile of the brain, steadily causing increased tumor necrosis factor alpha (TNF-α) levels, activated microglia, reduced brain-derived neurotrophic factor and DA levels [11]. Interestingly, chronic inflammation increased activity of monoamine oxidase B conferring susceptibility to 1-methyl-4-phenyl-1,2,3,6-tetrahydropyridine (MPTP) neurotoxic effect, which seems to be coordinated by microglia [12]. Although the precise mechanism is unclear, accumulating evidence points a pivotal role of systemic inflammation in the initiation and progression of PD.

Systemic inflammation is a crucial factor for glia activation in neurodegeneration [13], however, it is enigmatical where inflammation generates and produces. Our previous paper showed that p-α-syn aggregates for PD patients were found in Schwann cells (SCs) of sural nerves and triggered the release of inflammatory cytokines such as interleukin 1 beta (IL-1β), interleukin 6 (IL-6) and TNF-α [14]. SCs are the principal glia in PNS, where they provide allows for rapid nerve conduction by axons. The dysfunction of SCs is involved in the neuropathies of Charcot-Marie-Tooth disease, Guillain–Barré syndrome, schwannomatosis, and chronic inflammatory demyelinating polyneuropathy [15]. Current evidence suggests that SCs have the capacity to promote the repair and regeneration of multiple tissues [16]. Just like microglia, SCs also play an important role in immune surveillance, detecting pathogens, initiating and regulating local immune responses [17]. Nevertheless, the underlying mechanism of SCs mediating peripheral neuroinflammation and p-α-syn accumulation in PD peripheral neuropathy remains to be further explored.

PD peripheral neuropathies are associated with a variety of clinical symptoms ranging from motor and sensory symptoms to autonomic dysfunction, which are more common to occur in lower limbs than upper limbs [18]. Consistent with CNS, peripheral nerves exhibit degeneration and functional changes [19]. Sciatic nerve is the thickest nerve in lower limbs and contains sensory and motor nerve fibers, which preferably reflects peripheral neuropathy. Therefore, we used MPTP-induced chronic mouse model of PD and focused on the sciatic nerves to explore whether p-α-syn is aggregated in the glia of PNS and how SCs mediate neuroinflammation contributing to PD pathology.

## Results

### Morphological changes and ultrastructural destruction of peripheral nerves following MPTP exposure

**Fig. 1:**
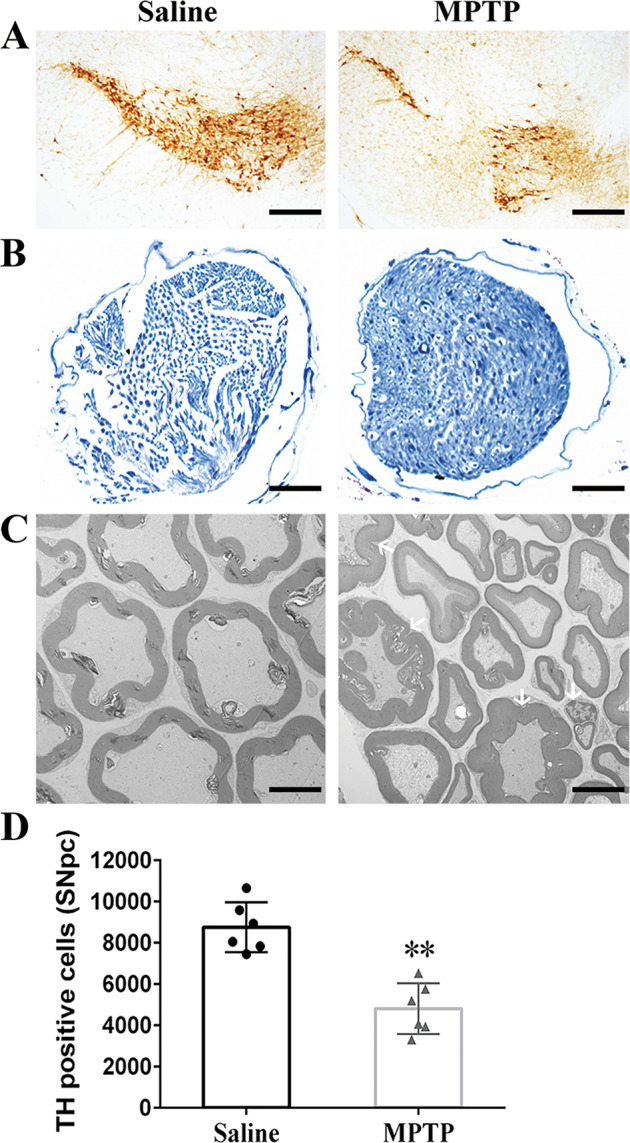
Morphological and ultrastructural destruction of sciatic nerves in MPTP-induced mice. **A** Representative images of TH^+^ neurons in SNpc. Scale bar, 200 µm. **B** Toluidine blue staining showed degenerated myelin sheath and atrophied axons in sciatic nerves of MPTP group compared with saline group. Scale bar, 200 µm. **C** Electron micrographs of myelinated axons suggested that homogeneous SCs and axons were neatly arranged in sciatic nerves of saline group, with swollen myelin layer, fragmentized SCs and accumulated degenerative products in MPTP group. Scale bar, 2 µm. **D** Stereological quantitative analysis displayed decreased TH^+^ neurons in SNpc after MPTP exposure. Data were presented as mean ± SEM and analyzed by Student’s *t*-test; *n* = 6. **p < 0.01 vs. saline group. MPTP, 1-methyl-4-phenyl-1,2,3,6-tetrahydropyridine; TH, tyrosine hydroxylase; SNpc: substantia nigra pars compacta; SCs: Schwann cells; SEM: standard error of the mean.

A significant reduction of 45% (**p < 0.01) for TH^+^ neurons was observed in SNpc of MPTP group compared to saline group (Fig. 1A, D). For morphological changes of sciatic nerve, fibers in saline group appeared loose arrange due to nerve vulnerability and the shape of SCs and axons were clearly visible; in contrast, a majority of axons shrank and even formed vacuoles in MPTP group, with swollen and fragmentized SCs (Fig. 1B). Furthermore, we used transmission electron microscope (TEM) to observe ultrastructural changes of sciatic nerve and found that myelinated and unmyelinated fibers were both contained. In saline group, homogeneous SCs and axons were neatly arranged, where SC myelin was compacted into a multilayer structure. However, swollen even demyelinated SCs and degenerative axons of sciatic nerve were noted in MPTP group (Fig. 1C).

### Expression of p-α-syn in midbrain and SCs of sciatic nerves after MPTP injection

**Fig. 2:**
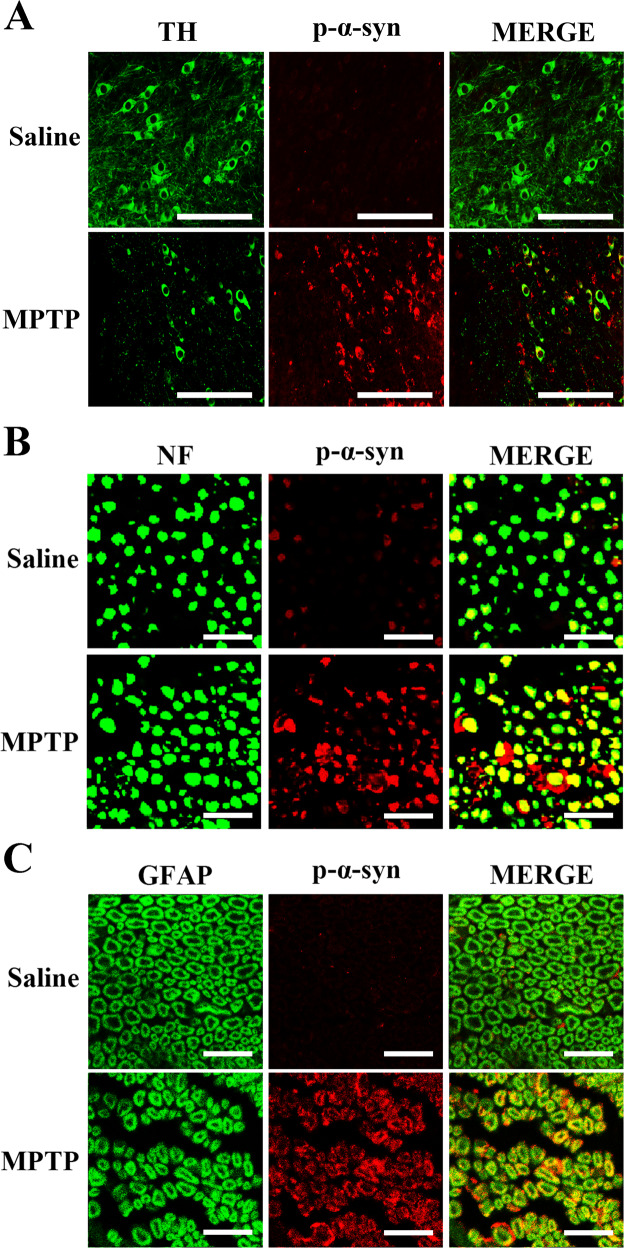
Immunofluorescence co-localization of TH with p-α-syn in midbrain, as well as p-α-syn with NF and GFAP in sciatic nerves of MPTP-induced mice. **A** A remarkable decline of TH^+^ neurons co-localized with p-α-syn was noted in SNpc of MPTP group compared to saline group; p-α-syn was located in the cytosol, the soma of TH^+^ neurons or fibers of non-TH^+^ neurons. Scale bar, 1 µm. **B** The p-α-syn in sciatic nerves of MPTP group was significantly increased than saline group. NF was used to label axons, showing dotted or granular; p-α-syn was expressed as puncta, ring or liner shape, partly in axons. Scale bar, 2 µm. **C** GFAP was a marker of SCs in myelin sheath showing ring or half ring shape around axons; p-α-syn was mainly distributed in SCs. Scale bar, 2 µm. TH: tyrosine hydroxylase; p-α-syn: phosphorylated α-synuclein; NF: neurofilament 200 Kd; GFAP: glial fibrillary acidic protein; MPTP, 1-methyl-4-phenyl-1,2,3,6-tetrahydropyridine; SCs: Schwann cells.

As is shown in Fig. 2A, p-α-syn in SNpc of MPTP group was elevated with the reduction of TH as compared with saline group. For sciatic nerves, p-α-syn was markedly increased as puncta, ring or liner shape in MPTP-treated mice. Further, neurofilament 200 Kd (NF) was all granular surrounded by p-α-syn, while glial fibrillary acidic protein (GFAP) in ring or half ring shapes was co-localized precisely with p-α-syn (Fig. 2B, C).

### Upregulated TLRs implicated with PD peripheral neuropathy from RNA-sequencing analysis

**Fig. 3:**
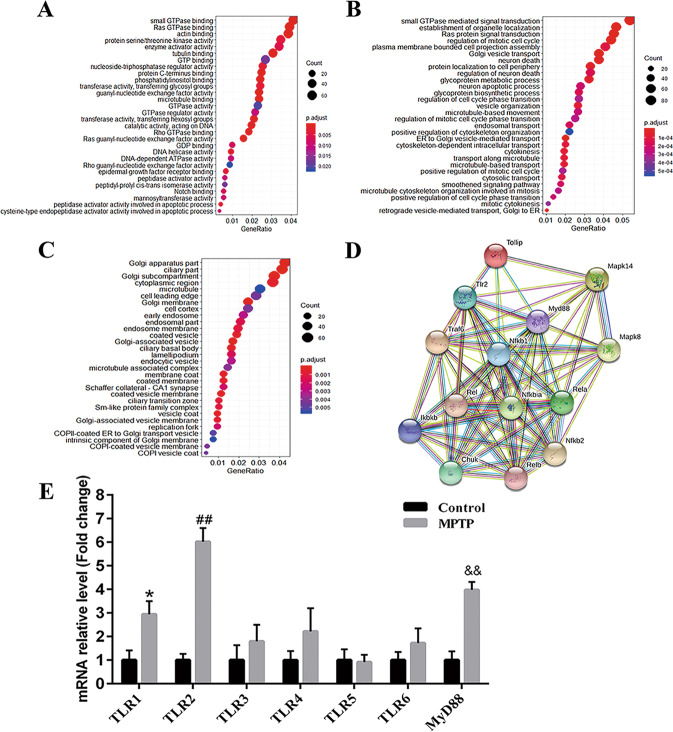
RNA-sequencing analysis of sciatic nerves showing TLRs implicated with PD peripheral neuropathy. **A** GO molecular function analysis. **B** GO biological process analysis. **C** GO cell composition analysis. **D** Related genes in TLRs signaling pathway. **E** qPCR validation. Data were presented as mean ± SEM and analyzed by one-way ANOVA followed by Tukey’s post hoc test for Fig. 3A–C; *n* = 4. Data in Fig. 3E were analyzed by Student’s *t*-test; *n* = 6. *p < 0.05, ^##^*p* < 0.01, and ^&&^*p* < 0.01 vs. saline group. MPTP: 1-methyl-4-phenyl-1,2,3,6-tetrahydropyridine; GO: Gene Ontology; qPCR: quantitative real-time polymerase chain reaction; TLR: toll-like-receptor; TLRs: toll-like receptors; MyD88: myeloid differentiation-factor 88; SEM: standard error of the mean; ANOVA: analysis of variance.

Differentially expressed genes (DEGs) were screened based on the following criteria: −2 < log2 (MPTP/saline) < 2 and *Q*-value < 0.05. A total of 715 genes were differentially expressed in MPTP-exposed mice from saline group, including 389 upregulated genes and 326 downregulated genes. Gene Ontology (GO) enrichment analysis revealed that DEGs were enriched in neuron death and regulation, protein serine/threonine kinase activity, microtubule movement, Ras protein signal transduction, and small GTPase signal transduction (Fig. 3A–C). Inflammation signaling network of DEGs suggested that 11 upregulated and 3 downregulated genes were closely linked with TLRs pathway (Fig. 3D), which was consistent with researches about connections between PD and neuroinflammation [20–22]. Then, TLR-related genes were selected for further confirmation using quantitative real-time polymerase chain reaction (qPCR), showing that TLR1 (*p < 0.05), TLR2 (^##^*p* < 0.01), and MyD88 (^&&^*p* < 0.01) had dominant expression pattern in sciatic nerves of MPTP group compared to saline group (Fig. 3E).

### Association of TLR2 with p-α-syn aggregated in SCs of sciatic nerves through TLR2/NF-κB pathway

**Fig. 4:**
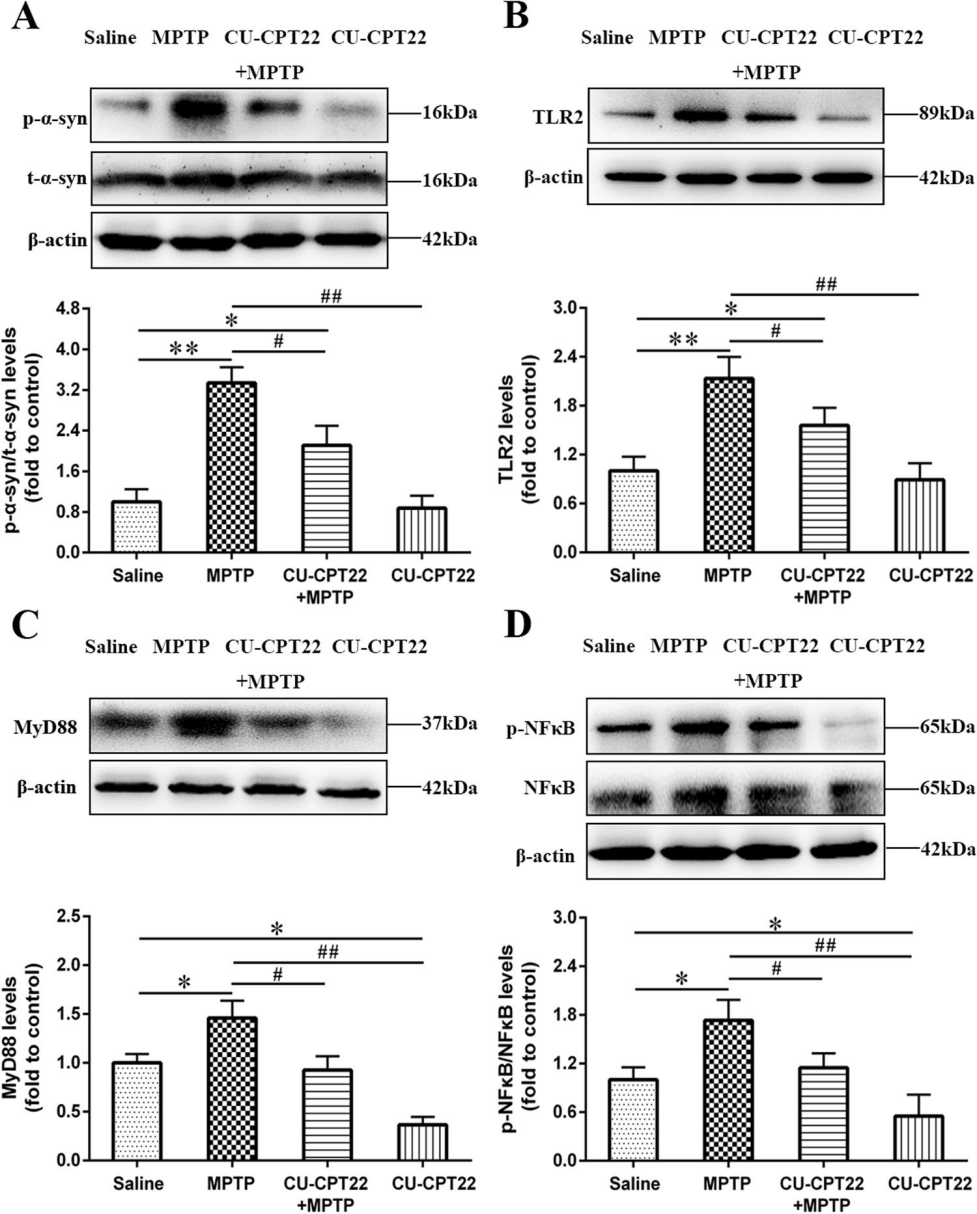
Accumulation of p-α-syn in SCs probably mediated by TLR2 through TLR2/NF-κB pathway. As a TLR2 inhibitor, CU-CPT22 was used to block TLR2/NF-κB pathway. **A** Level of p-α-syn was remarkably increased after MPTP exposure (20 mg/kg) and moderately elevated after CU-CPT22 + MPTP treatment (3 mg/kg; 20 mg/kg), while t-α-syn had no obvious changes. **B** Compared to saline group, the expression of TLR2 was higher in sciatic nerves for MPTP group and mildly upregulated in CU-CPT22 + MPTP group. **C** MyD88 in sciatic nerves of MPTP group was elevated than saline group, and level in CUCPT22 + MPTP group displayed a decrease compared with MPTP group. **D** The expression of p-NF-κB was higher in sciatic nerves of MPTP group than saline group, with a reduction in CUCPT22 + MPTP group compared to MPTP group. The β-actin was used as housekeeping and data were normalized to β-actin expression. Data were presented as mean ± SEM and analyzed by one-way ANOVA followed by Tukey’s post hoc test; *n* = 6. *p < 0.05, **p < 0.01 vs. saline group; ^#^*p* < 0.05, ^##^*p* < 0.01 vs. MPTP group. p-α-syn: phosphorylated α-synuclein; SCs: Schwann cells; MPTP: 1-methyl-4-phenyl-1,2,3,6-tetrahydropyridine; t-α-syn: total α-synuclein; TLR: toll-like-receptor; MyD88: myeloid differentiation-factor 88; NF-κB: nuclear factor kappa-B; p-NF-κB: phospho-NF-κB; SEM: standard error of the mean; ANOVA: analysis of variance.

To explore the underlying involvement of TLR2 in PD peripheral neuropathy, CU-CPT22, a TLR2 antagonist, was used. Western blotting analysis demonstrated that long-term MPTP administration effectively activated p-α-syn in sciatic nerves (**p < 0.01), while p-α-syn in CU-CPT22 + MPTP group showed less increase (^#^*p* < 0.05). However, the expression of total α-syn (t-α-syn) had no differences in four groups (Fig. 4A), suggesting that p-α-syn was the main pathology in PD peripheral neuropathy.

**Fig. 5:**
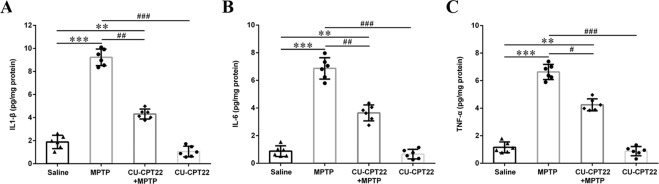
Elevation of inflammatory response in peripheral neuropathy of sciatic nerves in MPTP group, with considerable relief after CU-CPT22 treatment. **A** ELISA analysis of IL-1β in sciatic nerves of saline, MPTP, CU-CPT22 + MPTP and CU-CPT22 groups. **B** ELISA analysis of IL-6 in sciatic nerves of four groups. **C** ELISA analysis of TNF-α in sciatic nerves of four groups. Data were presented as mean ± SEM and analyzed by one-way ANOVA followed by Tukey’s post hoc test; *n* = 6. **p < 0.01, ***p < 0.001 vs. saline group; ^#^*p* < 0.05, ^##^*p* < 0.01, ^###^*p* < 0.001 vs. MPTP group. MPTP: 1-methyl-4-phenyl-1,2,3,6-tetrahydropyridine; ELISA: enzyme-linked immunosorbent assay; IL-1β: interleukin 1 beta; IL-6: interleukin 6; TNF-α: tumor necrosis factor alpha; SEM: standard error of the mean; ANOVA: analysis of variance.

**Fig. 6:**
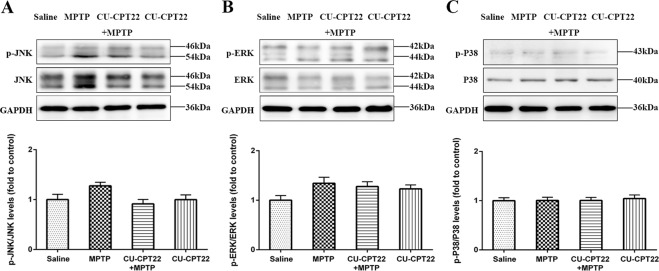
No remarked changes of other pathways (JNK, ERK, P38) in sciatic nerves after MPTP and CU-CPT22 challenging. **A** Western blotting analysis of p-JNK and JNK. **B** Western blotting analysis of p-ERK and ERK. **C** Western blotting analysis of p-P38 and P38. The β-actin was used as housekeeping and data were normalized to β-actin expression. Data were presented as mean ± SEM and analyzed by one-way ANOVA followed by Tukey’s post hoc test; *n* = 6. MPTP: 1-methyl-4-phenyl-1,2,3,6-tetrahydropyridine; JNK: c-Jun N-terminal kinase; p-JNK: phospho-JNK; ERK: extracellular regulated protein kinases; p-ERK: phospho-ERK; p-P38: phospho-P38; SEM: standard error of the mean; ANOVA: analysis of variance.

Compared to saline group, the expression of TLR2 (**p < 0.01), MyD88 (*p < 0.05) and p-NF-κB (*p < 0.05) was upregulated in sciatic nerves of MPTP group. After CU-CPT22 administration, the level of TLR2 (^#^*p* < 0.05), MyD88 (^#^*p* < 0.05) and p-NF-κB (^#^*p* < 0.05) was suppressed in CU-CPT22 + MPTP group compared to MPTP group (Fig. 4B–D). Enzyme-linked immunosorbent assay (ELISA) showed that the level of IL-1β (***p < 0.001), IL-6 (***p < 0.001) and TNF-α (***p < 0.001) was remarkably higher in MPTP group than saline group, with a significant recovery of IL-1β (^##^*p* < 0.01), IL-6 (^##^*p* < 0.01) and TNF-α (^#^*p* < 0.05) in CU-CPT22 + MPTP group compared to MPTP group (Fig. 5A–C). Proteins of other pathways including c-Jun N-terminal kinase (JNK), extracellular regulated protein kinases (ERK) and P38 and their phosphorylation exhibited no changes between four groups (Fig. 6A–C). Therefore, p-α-syn was mainly accumulated in SCs and probably activated SCs triggering a series of inflammatory responses through TLR2/NF-κB pathway.

### Motor performance and nerve conduction after MPTP and CU-CPT22 treatment

**Fig. 7:**
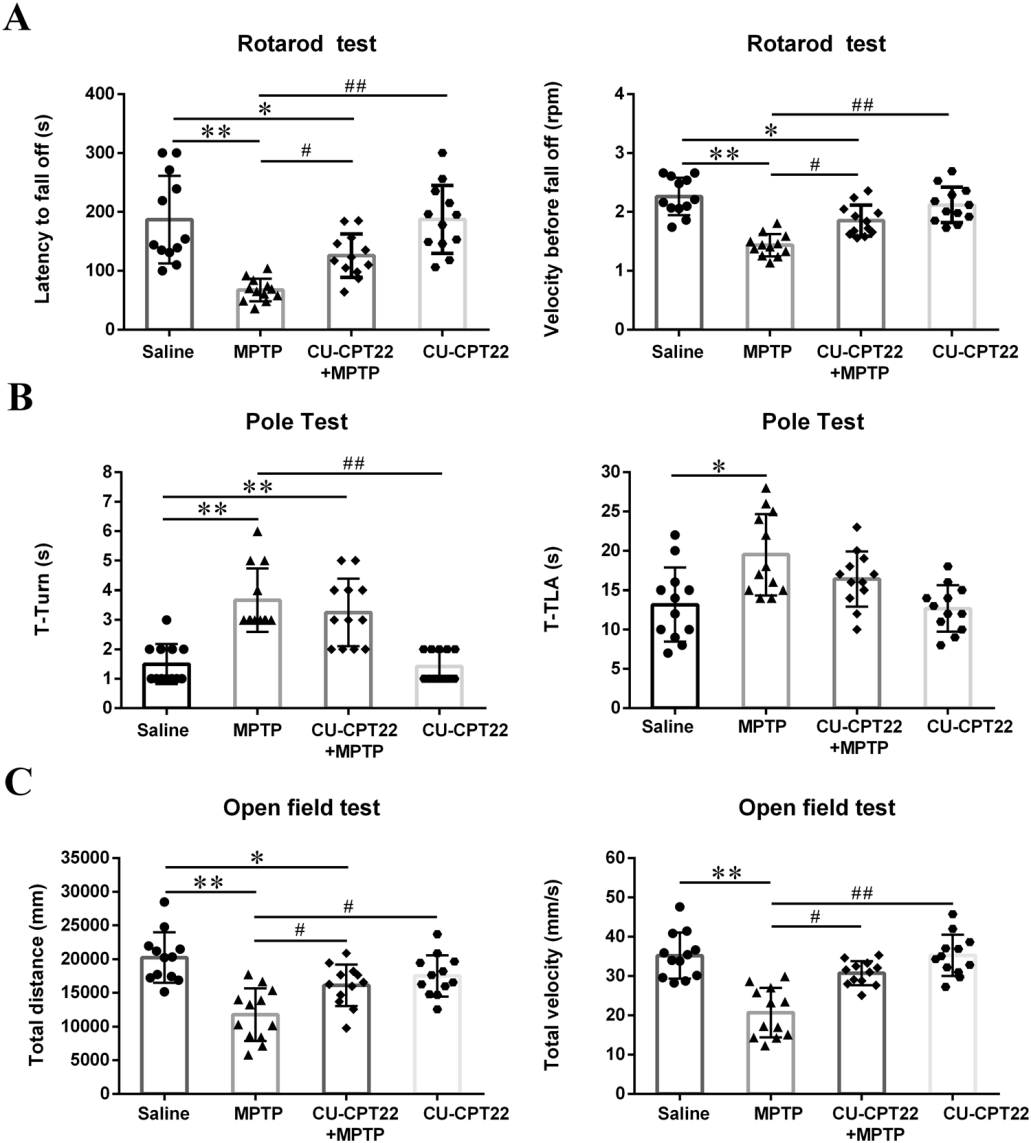
Motor dysfunction in MPTP-induced mice with improvement after CU-CPT22 preconditioning. Mice were tested 3 days after the last MPTP administration. **A** Latency to fall off and Velocity before fall off in Rotarod test. **B** T-turn and T-LA in Pole test. **C** Total distance and Total velocity in Open-field test. Data were presented as mean ± SEM and analyzed by one-way ANOVA followed by Tukey’s post hoc test; *n* = 12. *p < 0.05, **p < 0.01 vs. saline group; ^#^*p* < 0.05, ^##^*p* < 0.01 vs. MPTP group. MPTP: 1-methyl-4-phenyl-1,2,3,6-tetrahydropyridine; T-turn: time to turn; T-LA: time to climb down; SEM: standard error of the mean; ANOVA: analysis of variance.

During the dividing and exposing procedures, four mice were sacrificed because of unavoidable surgical trauma. Stay time on rotarod (**p < 0.01) was significantly reduced and mice could fall off the rotarod easily (**p < 0.01) in MPTP group compared to control mice, while CU-CPT22 + MPTP group exhibited longer stay time (^#^*p* < 0.05) and faster velocity (^#^*p* < 0.05) before fall off compared to MPTP group (Fig. 7A). Time to turn (T-turn) (**p < 0.01) and time to climb down (T-LA) (*p < 0.05) time in MPTP group were longer than saline group (Fig. 7B). For open-field test, MPTP group tended to move for shorter distance (**p < 0.01) at slower velocity (**p < 0.01) compared to control mice, while both distance (^#^*p* < 0.05) and velocity (^#^*p* < 0.05) improved in CU-CPT22 + MPTP group compared to MPTP group (Fig. 7C).

**Fig. 8:**
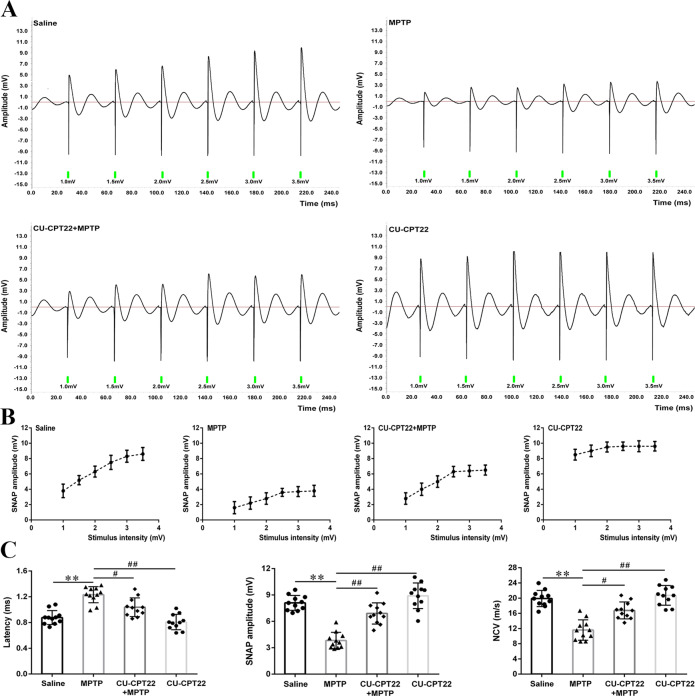
Electrophysiological damage of sciatic nerves in MPTP-administrated mice with amelioration in CU-CPT22 + MPTP group. Mice were tested 4 days after the last MPTP administration. **A** Representative electrophysiology of sciatic nerves for saline, MPTP, CU-CPT22 + MPTP and CU-CPT22 groups. **B** Stimulus-amplitude response curve in the four groups. **C** Quantitative analysis of latency, SNAP amplitude and NCV under a certain stimulus (3.5 mV) in the four groups. Data were presented as mean ± SEM and analyzed by one-way ANOVA followed by Tukey’s post hoc test; *n* = 11. **p < 0.01 vs. saline group; ^#^*p* < 0.05, ^##^*p* < 0.01 vs. MPTP group. MPTP: 1-methyl-4-phenyl-1,2,3,6-tetrahydropyridine; SNAP: sensory nerve action potential; NCV: nerve conduction velocity; SEM: standard error of the mean; ANOVA: analysis of variance.

Electrophysiological measurements were adopted to explore sensory neuropathy. Compared to saline group, sensory nerve action potential (SNAP) of sciatic nerves in MPTP group displayed small amplitudes and no significant alterations following voltage stimulation (Fig. 8A, B). Furthermore, the SNAP amplitudes of CU-CPT22 + MPTP group fell in between saline and MPTP groups, while CU-CPT22 group implicated a high amplitude under the low stimulus intensity and showed slight elevations with the stimulus gradually increasing (Fig. 8A, B), indicating the underlying protection effect of CU-CPT22 on the nerve conduction function of sciatic nerve. Postponed latency (**p < 0.01), reduced SNAP amplitude (**p < 0.01) as well as decreased nerve conduction velocity (NCV) (**p < 0.01) were emerged in MPTP group compared with saline group, while shortened latency (^#^*p* < 0.05), increased SNAP amplitude (^##^*p* < 0.01) and elevated NCV (^#^*p* < 0.05) were observed in CU-CPT22 + MPTP group compared with MPTP group (Fig. 8C).

## Discussion

One of PD pathological damages is p-α-syn aggregates [10], and abnormal accumulation of p-α-syn was detected in CNS and PNS [23]. Our prior study showed that p-α-syn was deposited in sural nerve of PD patients with a percentage of 100.0% rather than control subjects [24]. Role of p-α-syn has been established in peripheral nerves, whereas its underlying pathogenesis in PD requires additional investigation.

To determine PD peripheral neuropathy, two groups of systemic saline and MPTP administration were first utilized to resemble healthy control and PD model focusing on pathology of sciatic nerves. Toluidine blue staining showed degenerated myelin sheath and atrophied axons in sciatic nerves of MPTP mice, with electron micrographs capturing swollen myelin layer and fragmentized SCs, which implicate remarked SCs destruction in sciatic nerves following MPTP exposure. Compared with saline group, p-α-syn was mainly deposited in SCs of sciatic nerves for MPTP group (Fig. 2B, C). Then, RNA-sequencing analysis was adopted to investigate the underlying pathways involved and found that some upregulated and downregulated genes were closely linked with TLRs pathway (Fig. 3A–D). Outcomes from qPCR verified dominant expression of TLR1, TLR2 and MyD88 in sciatic nerves of MPTP group, with TLR2 expressing more than TLR1 (Fig. 3E).

As the principal glia of PNS, SCs function in nutrition, nerve regeneration and immunomodulation. Upon damage, SCs adopt a macrophage-like phenotype and return to a proliferating state to secrete chemokines, cytokines and neurotrophic growth factors, aiding in myelin phagocytosis [25, 26]. A research on A53T mice model implicated that peripheral neuroinflammation contributes to activated α-syn oligomers and exacerbated cognitive impairments [27]. Massive studies have revealed that TLRs are upregulated in neurodegenerative diseases such as Alzheimer’s disease, amyotrophic lateral sclerosis and PD [28–31]. It is reported that TLR2 is increased in the brain of PD patients and correlates with pathological α-syn [32]. In rodents, overexpression of α-syn promoted TLR2 expression and microglial activation [30, 33, 34]. TLRs can recognize different pathogen-associated molecular patterns and damage-associated molecular patterns triggering downstream cascades [34, 35]. Except TLR3, all TLR signaling pathways are dependent on MyD88, accompanying NF-κB phosphorylation and inflammatory response [36]. Previous reports have highlighted that TLRs are functional in SCs and bacterial lipoprotein yields the strongest response [37]. NF-κB plays a crucial role in neuroinflammation and initiates the transcription of pro-inflammatory gene coding for cytokines [36, 38]. A research on dementia with Lewy bodies addressed that NF-κB was involved in p-α-syn-mediated DA neuronal death [39].

Evidence suggests that some nonsteroidal anti-inflammatory drugs could function in the pathogenesis of synucleinopathies [15, 18, 40, 41]. To determine the exact mechanism of TLR2 pathway in PD peripheral neuropathy, CU-CPT22 was utilized in the following study. After CU-CPT22 treatment, accumulated p-α-syn and activated TLR2-mediated signaling were alleviated in CU-CPT22 + MPTP group when compared with MPTP group (Figs. 4 and 5), which was in consistent with our published study regarding the association between SCs and inflammatory cytokines in sural nerves of PD patients [14]. A research from Daniele et al. [30] demonstrated that CU-CPT22 might inhibit nuclear translocation of NF-κB and secretion of TNF-α in primary mouse microglia. Thus, MPTP-exposed mice in this work ideally exhibited PD peripheral neuropathy, indicating that p-α-syn aggregated in peripheral nerves was possibly involved in TLR2/ NF-κB pathway. Results from behavioral test illuminated that MPTP exposure caused motor performance dysfunction and CU-CPT22 might help to improve them (Fig. 7), suggesting that p-α-syn in sciatic nerves might cause motor nerve impairment and attenuated motor ability. These results were conformed to a study showing that systemic preconditioning with CU-CPT22 on MPTP mice protected motor nerve fibers and improved motor ability [13]. Additionally, MPTP mice performed postponed latency, reduced SNAP amplitude and decreased NCV than saline group from electrophysiology (Fig. 8), indicating degenerated sensory nerve fibers and blocked nerve conduction after MPTP exposure. Nevertheless, improved latency, SNAP amplitude and NCV were noticed in CU-CPT22 + MPTP group compared with MPTP group. Attenuated surgery-induced cognitive impairment in mice after CU-CPT22 injection might be seen in a recently published article [42]. Consistently, previous studies also demonstrated the improvement of nerve conduction in sciatic nerve after other therapies with anti-inflammatory properties [43, 44].

The current study is the first demonstrating p-α-syn aggregated in SCs of peripheral nerves has an influence on neuroinflammation probably related to TLR2/NF-κB signaling, which impairs motor ability and inhibits nerve conduction in MPTP-induced mice. Furthermore, our study extends our understanding on TLRs signaling and sheds light on potential new therapeutic avenues for PD.

However, there are some limitations in our research. Firstly, it is unclear whether p-α-syn is firstly deposited in the peripheral nerves or brain. Traditionally, p-α-syn was hypothesized to spread from PNS to the brain via vagus nerve [45]. Recent study proposed a route for long-distance bidirectional transmission of endogenous α-syn between PNS and CNS [46]. Therefore, p-α-syn aggregated in SCs may involve the origin and spread of PD pathology, which is worthwhile to be further studied. Secondly, TLR2 knockout mice or TLR2 siRNA treated mice will provide more conclusive evidence to validate the target role of TLR2 in PD peripheral neuropathy. Finally, the interaction between p-α-syn and TLR2 needs to be further confirmed in SCs due to the complexity in vivo.

## Materials and methods

### Experimental animals and model preparation

Four-month-old male C57BL/6J mice were acquired from the Animal Core Facility of Nanjing Medical University and were adapted to the experimental environment (12 h light–dark cycle, 22 °C ± 2 °C, 50–60% humidity) with ad libitum access to standard chow diet. Mice were first randomly divided into two groups: (1) MPTP (20 mg/kg, supplier seen in Table S1) was injected subcutaneously for MPTP group. After 1 h, the probenecid (250 mg/kg, supplier seen in Table S1) was intraperitoneally injected. (2) Saline group received a same dose of sterile saline and probenecid. The procedure lasted for 5 weeks with an interval of 3.5 days. To investigate the role of TLR2 in PD peripheral neuropathy, CU-CPT22, a TLR2 antagonist, was used for the following studies and mice were randomized into four groups (saline, MPTP, CU-CPT22 + MPTP and CU-CPT22 groups). CU-CPT22 (3 mg/kg; supplier seen in Table S1) was intraperitoneally administrated 30 min prior to MPTP injection in CU-CPT22 + MPTP group or solely for CU-CPT22 group. Mice were sacrificed 7 days after the last administration.

All experiments were approved and licensed by Ethical Committee of Nanjing Medical University and in accordance with the Guidelines for the Ethical Treatment of Experimental Animals issued by the Ministry of Science and Technology of the People’s Republic of China.

### Motor performance tests

Motor performance tests including rota-rod test, pole test and open-field test were conducted 3 days after the last administration to evaluate the motor, balance and coordination abilities [28, 47, 48].

### Electrophysiology

Mice were anesthetized by isoflurane inhalation (induction 3.5%, maintenance 1.5%). Sciatic nerves were stimulated percutaneously through a pair of needle electrodes at the sciatic notch (proximal site) and the ankle (distal site) [49–51]. Rectangular electrical pulses of 0.01 ms duration were applied up to different voltages from 0.5 to 3.5 mV, so that the sciatic nerves could give a maximal response. Latency, NCV and SNAP amplitudes were recorded with BL420 biological and functional experimental system (Taimeng Software Co., Ltd. Chengdu, China). Nerves were perfused with paraffin oil and ambient temperature was maintained at 37 °C with a pH of 7.4.

### Immunohistochemistry and immunofluorescence staining

Immunostaining method was described in a previous publication [52]. Detail information about the reagents in this part was shown in Table S1. Primary antibodies including anti-TH, anti-p-α-syn, anti-NF, and anti-GFAP were used either for midbrain or sciatic nerve. Then, secondary antibodies of Alexa Fluor 488 and 594 were incubated. Olympus FV1000 confocal laser scanning microscope was applied to acquire images.

For immunohistochemistry, brain slices were incubated with primary antibody of anti-TH. Number of TH^+^ neurons in SNpc of midbrain was assessed using optical fractionator (Stereo Investigator software, Microbrightfield Bioscience, Williston, VT, USA). All stereological analyses were performed under × 200 magnification of Olympus BX52 microscope (Olympus America Inc., Melville, NY, USA).

### Toluidine blue staining and TEM

Toluidine blue staining and TEM assays were performed to observe morphological changes in sciatic nerves [24, 53]. Specimens were cut into sections of 70 nm thick and examined with TEM (JEM-1010, Tokyo, Japan).

### ELISA analysis

Concentration of IL-1β, IL-6, and TNF-α was, respectively, measured by IL-1β ELISA kits (EM001-96, ExCell Bio, China), IL-6 ELISA kits (EM004-96, ExCell Bio) and TNF-α ELISA kits (EM008-96, ExCell Bio) according to manufacturer’s instructions.

### RNA-sequencing analysis

RNA-sequencing analysis was conducted in Beijing Genomics institution Co., Ltd. RNA samples with high purity (OD 260/280 ≥ 2.0) and integrity (RIN > 7.0) of sciatic nerves were used to construct the cDNA library (BGISEQ-500 platform). We got an average of 21.83 Mb clean reads and 18623 genes, with an average genome mapping rate of 95.17%. GO analysis was performed on 168 hub genes obtained from weighted gene co-expression network analysis and DEGs network was analyzed using an online tool (https://string-db.org/) [54]. The background was set to Mus musculus and the enrichment threshold was *p* < 0.05 (corrected Fisher exact *p-*value).

### qPCR analysis

Total RNA was extracted from sciatic nerves using Trizol reagent (Thermo Fisher Scientific) and converted to cDNA using reverse transcription kit (Takara, RR047A). Then, cDNA was used for qPCR (SYBR Premix Ex TaqII Kit) on ABI 7500 real-time PCR system (Applied Biosystems, Foster City, CA, USA) following the manufacturer’s protocol. Results were expressed as 2^−^^ΔΔCt^ and all primers were shown in Table S2.

### Western blotting analysis

According to an established protocol [55], the protein lysates of sciatic nerves were quantified by Bradford assays (Bio-Rad, Hercules, CA, USA). Reagents were displayed in Table S1. Average blot intensities were calculated using Image J, and values were reported as average intensity above the background, where the β-actin was used for normalization.

### Statistical analysis

In this study, the experiment implementation, data collection and statistical analysis were all completed based on the double-blind principle. Data were analyzed by Student’s *t*-test for individual comparisons between two groups using GraphPad Prism (GraphPad software, Inc., San Diego, CA). One-way analysis of variance (ANOVA) followed by Tukey’s post hoc test was used for multiple groups. Data were expressed as mean ± standard error of the mean (SEM), and *p* < 0.05 was considered significant.

## Supplementary information

Table S1

Table S2

Author Contribution

## Data Availability

The datasets used and/or analyzed in this study are available from the corresponding author on reasonable request.
