# Supplementary material for: Phosphorylated α-synuclein aggregated in Schwann cells exacerbates peripheral neuroinflammation and nerve dysfunction in Parkinson’s disease through TLR2/NF-κB pathway
Source: Cell Death Discov. 2021 Oct 12;7:289. doi: 10.1038/s41420-021-00676-w (PMC8511120; doi:10.1038/s41420-021-00676-w)
Supplement: Supplementary file 1 — Table S1 [file 41420_2021_676_MOESM1_ESM.docx]

**Table S1 Information for treatments/antibodies.**

| **Treatments/antibodies** | **Supplier** | **Concentration** |
| --- | --- | --- |
| MPTP | Sigma-Aldrich,MO, USA | 20 mg/kg |
| Probenecid | Watson Pharma Inc., CA, USA | 250 mg/kg |
| CU-CPT22 | S8677, Selleck, USA | 3mg/kg |
| anti-TH | #AB152, Sigma-Aldrich, USA | IF-1:2000; IHc-1:1000 |
| anti-p-α-syn | pSyn#64, WAKO, JAPAN | IF-1:500; WB-1:2000 |
| anti-NF | #ab207176, Abcam, UK | 1:100 |
| anti-GFAP | #ab33922, Abcam, UK | 1:500 |
| Alexa Fluor 488 | #ab150077, Abcam, UK | 1:400 |
| Alexa Fluor 594 | #ab150108, Abcam, UK | 1:400 |
| anti-α-syn | #ab1903, Abcam, UK | 1:4000 |
| anti-TLR2 | #ab209216, Abcam, UK | 1:1000 |
| anti-MyD88 | #4283, Cell Signaling, USA | 1:1000 |
| anti-p-NF-κB | #3033, Cell Signaling, USA | 1:1000 |
| anti-NF-κB | #8242, Cell Signaling, USA | 1:1000 |
| anti-p-JNK | #9255, Cell Signaling, USA | 1:1000 |
| anti-JNK | #9252, Cell Signaling, USA | 1:1000 |
| anti-p-ERK | #4370, Cell Signaling, USA | 1:1000 |
| anti- ERK | #4695, Cell Signaling, USA | 1:1000 |
| anti-p-P38 | #4511, Cell Signaling, USA | 1:1000 |
| anti-P38 | #8690, Cell Signaling, USA | 1:1000 |
| anti-β-actin | #66009-1-Ig, Proteintech, USA | 1:5000 |

**Abbreviations:** MPTP: 1-methyl-4-phenyl-1,2,3,6-tetrahydropyridine; TH: tyrosine hydroxylase; p-α-syn: phosphorylated α-synuclein; NF: neurofilament 200Kd; GFAP: glial fibrillary acidic protein; α-syn: α-synuclein; TLR: toll-like-receptor; MyD88: myeloid differentiation-factor 88; NF-κB: nuclear factor kappa B; p-NF-κB: phospho-NF-κB; JNK: c-Jun N-terminal kinase; p-JNK: phospho-JNK; ERK: extracellular regulated protein kinases; p-ERK: phospho- ERK; p-P38: phospho-P38.
