# Supplementary material for: Phosphorylated α-synuclein aggregated in Schwann cells exacerbates peripheral neuroinflammation and nerve dysfunction in Parkinson’s disease through TLR2/NF-κB pathway
Source: Cell Death Discov. 2021 Oct 12;7:289. doi: 10.1038/s41420-021-00676-w (PMC8511120; doi:10.1038/s41420-021-00676-w)
Supplement: Supplementary file 2 — Table S2 [file 41420_2021_676_MOESM2_ESM.docx]

**Table S2 Primers for qPCR.**

| **Gene ID** | **Forward primer** | **Reverse primer** |
| --- | --- | --- |
| TLR1 | TGAGGGTCCTGATAATGTCCTAC | AGAGGTCCAAATGCTTGAGGC |
| TLR2 | CCACTGCCCGTAGATGAAGT | CCCATTGAGGGTACAGTCGT |
| TLR3 | GTGAGATACAACGTAGCTGACTG | TCCTGCATCCAAGATAGCAAGT |
| TLR4 | AAATGCACTGAGCTTTAGTGGT | TGGCACTCATAATGATGGCAC |
| TLR5 | GCAGGATCATGGCATGTCAAC | ATCTGGGTGAGGTTACAGCCT |
| TLR6 | TGAGCCAAGACAGAAAACCCA | GGGACATGAGTAAGGTTCCTGTT |
| MyD88 | TCATGTTCTCCATACCCTTGGT | AAACTGCGAGTGGGGTCAG |
| GAPDH | AACGACCCCTTCATTGAC | TCCACGACATACTCAGCAC |

**Abbreviations:** qPCR: quantitative real-time polymerase chain reaction; ID: identification; TLR: toll-like-receptor; MyD88: myeloid differentiation-factor 88; GAPDH: glyceraldehyde-phosphate dehydrogenase.
