# Supplementary material for: Phosphorylated α-synuclein aggregated in Schwann cells exacerbates peripheral neuroinflammation and nerve dysfunction in Parkinson’s disease through TLR2/NF-κB pathway
Source: Cell Death Discov. 2021 Oct 12;7:289. doi: 10.1038/s41420-021-00676-w (PMC8511120; doi:10.1038/s41420-021-00676-w)
Supplement: Supplementary file 3 — Author Contribution [file 41420_2021_676_MOESM3_ESM.pdf]

DECLARATION OF CONTRIBUTIONS TO ARTICLE

ADMC

Manuscript Number:	Journal Name:	
CDDISCOVERY-21-1464R2	*Cell Death Discovery*	(the 'Journal')

Proposed Title of the Contribution:

Phosphorylated α-synuclein aggregated in Schwann cells exacerbates peripheral neuroinflammation and nerve dysfunction in Parkinson's disease through TLR2/NF-κB pathway (the 'Contribution)

Author(s):

Li Sun, Wenwen Jiang, Ye Wang, Yongsheng Yuan, Zhe Rong, Jing Wu, Yi Fan, Ming Lu and Kezhong Zhang (the 'Authors')

For all *CDDis*covery articles, each person named as an author in the published version must be able to show he or she has contributed substantially to the article.

Authorship credit should be based on 1) substantial contributions to conception and design, acquisition of data, or analysis and interpretation of data; 2) drafting the article or revising it critically for important intellectual content; and 3) final approval of the version to be published. Authors should meet conditions 1, 2 and 3.

Any person who cannot be shown to have made a substantial contribution to the article cannot be listed as an author in the final version. The name of any person who is deemed to have made a minor contribution can, however, appear in the Acknowledgments section of the article.

Please complete the table below to indicate the contributions of all named authors to the manuscript.

Author Full Name:	Specification of Contribution to the Manuscript:
Li Sun	study design, performing experimentations, data analysis, writing and revising the manuscript.
Wenwen Jiang	study design, performing experimentations, data analysis, writing and revising the manuscript.
Ye Wang	study design, performing experimentations, data analysis, writing and revising the manuscript.
Yongsheng Yuan	study design and obtaining fundings.
Zhe Rong	study design, performing experimentations and data analysis.
Jing Wu	study design, performing experimentations and data analysis.
Yi Fan	study guidance and supervision.
Ming Lu	study guidance and supervision, revising the manuscript.
Kezhong Zhang	study design and supervision, obtaining fundings, revising the manuscript.

Please complete the table below to indicate the contributions of all named authors to the figures.

Figure 1:

L.S., W.J., Y.W. , Z.R., and J.W. performed the experiments, acquired the images, analyzed the data, made bar diagrams and produced the figure. K.Z., M.L. Y.F. and Y.Y. guided and supervised the figure production.

Figure 2:

L.S., W.J., Y.W. , Z.R., and J.W. performed the experiments, acquired the images, analyzed the data, made bar diagrams and produced the figure. K.Z., M.L. Y.F. and Y.Y. guided and supervised the figure production.

Figure 3:

L.S., W.J., Y.W. , Z.R., and J.W. performed the experiments, acquired the images, analyzed the data, made bar diagrams and produced the figure. K.Z., M.L. Y.F. and Y.Y. guided and supervised the figure production.

Figure 4:

L.S., W.J., Y.W. , Z.R., and J.W. performed the experiments, acquired the images, analyzed the data, made bar diagrams and produced the figure. K.Z., M.L. Y.F. and Y.Y. guided and supervised the figure production.

Figure 5:

L.S., W.J., Y.W. , Z.R., and J.W. performed the experiments, acquired the images, analyzed the data, made bar diagrams and produced the figure. K.Z., M.L. Y.F. and Y.Y. guided and supervised the figure production.

Figure 6:

L.S., W.J., Y.W. , Z.R., and J.W. performed the experiments, acquired the images, analyzed the data, made bar diagrams and produced the figure. K.Z., M.L. Y.F. and Y.Y. guided and supervised the figure production.

Signed for and on behalf of the Author(s):	Print Name:	Date:
Kezhong Zhang	Kezhong Zhang	Sep 23, 2021
